# Framing overdiagnosis in breast screening: a qualitative study with Australian experts

**DOI:** 10.1186/s12885-015-1603-4

**Published:** 2015-08-28

**Authors:** Lisa M. Parker, Lucie Rychetnik, Stacy Carter

**Affiliations:** 1 Centre for Values, Ethics and the Law in Medicine (VELiM), Sydney School of Public Health, The University of Sydney, Medical Foundation Building, K 25 (92-94 Parramatta Road), Sydney, NSW 2006 Australia; 2 School of Medicine Sydney, The University of Notre Dame, 160 Oxford St, Darlinghurst, NSW 2010 Australia

## Abstract

**Background:**

The purpose of this study was to identify how the topic of overdiagnosis in breast cancer screening is framed by experts and to clarify differences and similarities within these frames in terms of problems, causes, values and solutions.

**Methods:**

We used a qualitative methodology using interviews with breast screening experts across Australia and applying framing theory to map and analyse their views about overdiagnosis. We interviewed 33 breast screening experts who influence the public and/or policy makers via one or more of: public or academic commentary; senior service management; government advisory bodies; professional committees; non-government/consumer organisations. Experts were currently or previously working in breast screening in a variety of roles including clinical practice, research, service provision and policy, consumer representation and advocacy.

**Results:**

Each expert used one or more of six frames to conceptualise overdiagnosis in breast screening. Frames are described as: Overdiagnosis is harming women; Stop squabbling in public; Don’t hide the problem from women; We need to know the overdiagnosis rate; Balancing harms and benefits is a personal matter; and The problem is overtreatment. Each frame contains a different but internally coherent account of what the problem is, the causes and solutions, and a moral evaluation. Some of the frames are at least partly commensurable with each other; others are strongly incommensurable.

**Conclusions:**

Experts have very different ways of framing overdiagnosis in breast screening. This variation may contribute to the ongoing controversy in this topic. The concept of experts using different frames when thinking and talking about overdiagnosis might be a useful tool for those who are trying to negotiate the complexity of expert disagreement in order to participate in decisions about screening.

**Electronic supplementary material:**

The online version of this article (doi:10.1186/s12885-015-1603-4) contains supplementary material, which is available to authorized users.

## Background

Overdiagnosis in breast screening has become a highly contentious issue and source of strong disagreement amongst experts. In this paper we use the term “overdiagnosis” to mean the diagnosis through mammographic screening of an asymptomatic breast condition that is non-progressive or so slowly progressive that it would not otherwise have come to the patient’s attention in her lifetime, and where this diagnosis provides no net benefit to the patient [1]. The possibility of overdiagnosis in breast screening was acknowledged from its early days of use. The idea that breast screening might lead to the detection of lesions that are “morphologically malignant but clinically benign” was raised as early as the 1970s ([2], p490). Later it was also recognised that mammographic screening would uncover a significant number of in-situ cancers, at least some of which “might not have entered an invasive phase during their lifetime” ([3], p14) and would likely fall into the category of overdiagnosis. Despite this, there was limited controversy about overdiagnosis when breast screening programs were being introduced in many Western countries during the 1980s and 1990s. This may have been partly because of poor outcomes from treatment of symptomatic breast cancers and the evidence-based promise of a 30 % reduction in population breast cancer mortality.

Since that time, however, the evidence-based estimates of the mortality benefit from breast screening have been revised and reduced [4, 5]. In addition, improvements in breast cancer treatment are likely to have further reduced the potential impact of screening in the modern Western setting [5, 6]. These developments have fostered a growing interest amongst breast screening experts about the significance of overdiagnosis, which is now a topic of major international concern [7–9].

Researchers and clinicians present many different views about overdiagnosis, and focus on different problems and solutions, including: preventing overdiagnosis harm [10]; communicating with women about overdiagnosis [11–13]; and quantification of overdiagnosis [14–16]. There are also big differences of opinion within these topics. Understanding how and why experts form their opinions about this complex issue, and sometimes arise at opposing views, would add to our understanding of the current processes for early detection in breast cancer and assist those who seek to contribute to mammography screening policy, as well as those participating in consumer decisions about screening.

We conducted a detailed qualitative study of the views and opinions of Australian breast screening experts on a range of topics related to mammography screening. We used a framing approach to map and analyse experts’ views on the issue of overdiagnosis. Framing describes the particular mind-set through which a topic is understood. The framing of an issue determines how the problem is conceived, what information is selected and the value judgements that are made. Different frames incorporate different, apparently self-evident, strategies to solve the perceived problem [17, 18]. Frames can be used in politics or by institutions to convey a particular message or point of view [19]. Frames are not only used as deliberate tools: they are also used by individuals, often unconsciously, as a way of thinking about and making sense of a complex topic. Framing theory is particularly well-suited to the study of overdiagnosis because it allows for a detailed examination of different viewpoints held, and used, by experts about this contentious topic. We present our analysis of how experts framed the topic of overdiagnosis in breast screening. Our research questions were:

- How do Australian breast screening experts frame overdiagnosis?
- How do those frames present the problems, causal elements, value judgements and solutions relevant to overdiagnosis?

## Methods

This study is part of a larger Australian National Health and Medical Research Council (NHMRC) funded project examining ethical issues in cancer screening in Australia [20]. One component of the larger project was a qualitative study of contemporary issues in breast cancer screening, using semi-structured interviews with influential breast screening experts. This paper is reporting on one aspect of this breast screening study. We defined “influential experts” as people working or researching in breast screening who influence the public, primary care practitioners and/or policy makers by engaging in one or more of: media commentary; academic or lay publications and presentations; senior service delivery management; membership of government advisory bodies, professional committees and/or non-government/consumer organisations related to breast screening. We sampled purposively from this population, seeking to obtain a wide diversity of views by inviting participants with a range of publicly aired positions [21]. We reasoned that perspectives on screening might be associated with professional backgrounds so we ensured that we included experts with a range of roles and responsibilities. See Table 1 for further participant details.

**Table 1:** Characteristics of experts

Participants 33 (Brackets contain number of experts who were invited but did not-participate; 13)	
Professional role*	Clinicians^ 15 (3)
	• Oncologists 3 (1)
	• Surgeons 4 (0)
	• Breast physicians 1 (2)
	• Radiologists 2 (0)
	• Radiation oncologists 2 (0)
	• Pathologists 3 (0)
	• Others [not otherwise specified; NOS)] 0 (1)
	Non-clinical researchers 14 (3)
	• Epidemiologists/biostatisticians 9 (1)
	• Others [NOS] 5 (1)
	Administrators/managers 6 (2)
	Advocacy leaders 6 (7)
	• Consumers working in advocacy 3 (6)
	• Clinicians/researchers working in advocacy 3 (1)
Public stance on breast screening+	Supportive 16 (9)
	Mostly supportive^#^ 3 (1)
	Critical 6 (0)
	Unknown to researchers 8 (3)

*note that some experts held more than one professional role

^Most clinicians engaged in research to a greater or lesser extent

^+^We loosely categorised potential interviewees as being “supportive”, “mostly supportive” or “critical” about breast screening based on publicly available commentary

^#^Broadly supportive of breast screening but with selected concerns about one or more elements of the program

We identified potential participants by scanning academic and lay literature on breast screening, examining personnel lists on websites of government or non-government advisory and advocacy bodies involved in breast screening, and following up suggestions from colleagues and participants. We used information in the public domain to contact experts by email. Forty-six experts were contacted, and 33 (17 male, 16 female) participated in the study. Thirteen people either did not wish to participate (3), did not respond (9) or were unable to participate in the time available (1). We had a low response rate from senior community advocacy figures. Speculatively, this may have been due to a higher turnover of staff in these (largely volunteer) positions than in other professional roles. That is, the individuals may no longer have been contactable at the email addresses that we had access to. We continued sampling until we had good representation of a range of professional roles and until we reached thematic saturation in our analysis [22].

We used an interview format for in-depth exploration of the views and reasoning of experts. LP conducted semi-structured interviews from October 2012 to October 2013, meeting in the participant’s or her own workplace, or talking over telephone if unable to meet in person. The interviews lasted between 39 and 105 min (average 66 min) and there was no observed difference between face to face and telephone interviews in terms of quality or length [23]. At the beginning of each interview, LP discussed her interest in the topic with the expert, explaining that she was a medical practitioner with clinical experience in breast screening, currently undertaking doctoral studies in cancer-screening ethics. She clarified that the purpose of the interviews was to glean the range of opinions amongst Australian experts about breast screening. The interviews drew loosely on a set of core questions designed to draw out the participant’s views. We also sought to tailor each interview to the particular expertise and interests of the participants, and explored the leads and topics that arose throughout the discussion [22, 24]. We encouraged the participants to talk about overdiagnosis, asking generally for interviewees’ views on this topic, without pre-empting ideas about what might be considered important. We only pursued particular lines of enquiry about controversial elements – as informed by the literature – if this flowed on from preceding comments of the participant. An additional file outlines sample interview questions (see Additional file 1).

The interviews were taped, transcribed and de-identified. We used an inductive analytic methodology, developing a set of categories that captured the most important views and values in the experts’ comments. Each interview was read repeatedly and coded in detail to capture views and values relevant to overdiagnosis. The analysis was conducted as an iterative process comprising detailed coding of individual transcripts (LP) and discussion and revision of the findings in group analysis meetings (all authors). We used framing theory to organise and understand different ways that experts thought about overdiagnosis, identifying the dominant frames in use and categorising important elements of each frame in terms of problems, causes, solutions and moral evaluation [18].

Ethics approval was granted from the Cancer Institute NSW Population & Health Services Research Ethics Committee [HREC/12/CIPHS/46] and the University of Sydney Human Research Ethics Committee [#15245]. All participants gave individual consent to be interviewed, and were free to withdraw from the study at any stage.

## Results

We identified six frames that Australian breast screening experts used with regard to overdiagnosis (Table 2).

**Table 2:** Overdiagnosis frames adopted by Australian breast screening experts

Frame	Defining the problem	The reasons for the problem	Value judgement	Proposed or implied solution
1. Overdiagnosis is harming women	Breast screening is resulting in significant harm to women because of overdiagnosis	The harms associated with overdiagnosis are significant in both quantity and quality	Breast screening programs should pay more attention to avoiding the serious harms of overdiagnosis	Reduce overdiagnosis either by performing targeted screening or by reducing screening overall
2. Stop squabbling in public about overdiagnosis	The public discussion of overdiagnosis is generating negative publicity which may reduce breast screening participation & is therefore a disservice to women	Exaggeration of harms in public debates is causing confusion amongst women and threatening participation rates.	Breast screening commentators should give priority to delivering health benefits (saving lives)	Confine discussion about overdiagnosis to academic circles only, avoiding public confusion
3. Don’t hide the overdiagnosis problem from women	The breast screening program is not facilitating informed choice amongst women	There is a deliberate lack of communication about overdiagnosis from breast screening providers because of a desire to maximise breast screening participation	Breast screening should give absolute priority to promoting autonomy via informed choice	Fully inform women about overdiagnosis
4. We need to know the overdiagnosis rate	It is not clear how much overdiagnosis is present in breast screening	There is huge variation in overdiagnosis rates due to different methodologies and/or data sets; differences in the way overdiagnosis figures are presented hampers interpretation by non-epidemiologists	Overdiagnosis research should be more rigorous, robust and consistent	Commit to reaching a consensus on appropriate methodology & the way we report the figures
5. Balancing harms and benefits is a personal matter	It is not clear how to compare the harms & benefits of breast screening	It is impossible for experts to definitively compare harms & benefits because they are qualitatively different	Breast screening decision making should be guided by a consumer-orientated process, which takes into account public attitudes to harms and benefits	Use deliberative methods to inform policy decisions; support individual consumers to make personal decisions about participation
6. The problem is overtreatment	Breast screening is resulting in overdiagnosis which leads to overtreatment of some women	Management of some women with cancer is sometimes unnecessarily aggressive because we don’t know enough about the natural history of screen detected lesions	While it is important that screening continues to save lives, we should seek ways to reduce harms from unnecessary (over) treatment	Ongoing education for pathologists; renaming non-invasive lesions; research into prognostic biomarkers, targeted treatments & less aggressive management regimes; patient centred care

### Frame 1: overdiagnosis is harming women

*“I would like to see breast cancer eradicated too but not at the expense of … potentially treating them with serious treatments for a condition that maybe didn’t need to be found in the first place… To me, it’s all about how do we run this program in a way that minimises the harm … without losing the benefit.”* (Expert #33, clinician)

Experts who used this frame were passionate about the topic of overdiagnosis in breast screening and saw it as a major threat to the wellbeing of women. The frame emphasised both quantity and quality of harm. Harm quantity was described in terms of the high number of overdiagnosed cases compared to the number of lives saved by screening. Harm quality was discussed by highlighting the serious negative impact from each case of overdiagnosis, including both the psychological impact of a breast cancer diagnosis on a woman and her female relatives (for whom it has perceived risk implications), and the short and long term impact of unnecessary treatment on lifestyle and physical health. This framing of overdiagnosis as a serious problem was grounded in a strong commitment to avoiding harm in any public health program.

This frame encompassed two categories of solution. Experts who were enthusiastic about the potential benefits of screening suggested reducing overdiagnosis through a targeted, personalised screening program, matching recommended screening frequency to breast cancer risk as determined by factors such as breast density. This would enable the population to simultaneously retain benefits of screening and reduce harms. Experts who were more sceptical about the benefits accruing from breast screening preferred a more extreme solution: reducing overdiagnosis by decreasing overall breast screening participation. However, they assumed that cessation of public funding for the program was politically unlikely, and promoted more realistic solutions such the removal of governmental promotions and personalised screening invitations.

### Frame 2: stop squabbling in public about overdiagnosis

*“I feel that it’s unwarranted … when … the [overdiagnosis] debate is mentioned in a way that it might deter people from actually participating in screening. I think that’s really counterproductive… The debate should be managed in a way that it’s not inadvertently discouraging screening.”* (Expert #10, consumer advocate)

This frame centres on the negative publicity generated by overdiagnosis discussions and the decrease in breast screening participation that might ensue. Underlying this concern is a firm belief in the net benefit of breast screening and a strong desire to have women avail themselves of life-saving opportunities. The frame delivers a choice between life and overdiagnosis: “*saving a life is more important than the harm that’s caused in damaging normal breasts.”* (Expert #3, clinician). Experts using this frame regarded overdiagnosis as a minor problem, for several reasons. Firstly, and most commonly, it was seen as an inevitable part of screening, particularly breast screening where cancer growth is variable and unpredictable. Secondly, the number of overdiagnosed cases was considered low relative to the total number of breast cancers picked up through the program. Finally, the harm associated with each overdiagnosed case was seen as low. This was justified in several ways: 1) individual women could not know whether or not their cancer was a case of overdiagnosis; 2) women (allegedly) disregarded the concept of overdiagnosis when considering treatment options; and 3) treatment for small, low-grade cancers (ie those most likely to be cases of overdiagnosis) was viewed as relatively benign. In addition to the lack of harm, the frame highlighted possible benefits from overdiagnosis. Although, by definition, an overdiagnosed cancer will not itself threaten a woman’s life, experts suggested that as the woman would be at increased risk of a second breast cancer she would benefit from being identified and treated with tamoxifen.

In this frame, personal autonomy and informed choice were important values in healthcare. However experts rejected the idea that stopping ‘squabbling in public’ might conflict with respecting womens’ autonomy. Their central concern was not so much that overdiagnosis was mentioned, but that overdiagnosis was invariably (mis) represented as an important harm:

*“Harm is a term that’s been developed by academics, along academic lines*… *There’s a possibility of over diagnosis … it’s not very much … you shouldn’t call that harmful.”* (Expert #17, consumer advocate)

Some experts used this frame with the view that informed choice was an unattainable goal, because overdiagnosis in breast screening is just so complex:

*“There’s all this business of informed consent. Well, frankly, I think it’s for the birds. I think it’s a very difficult thing for people to have informed consent. When people argue a lot, you know, people that are informed, supposedly, argue, I don’t know how you give informed consent. It’s very difficult for the average layperson to understand.”* (Expert #9, clinician)

There was also moral condemnation of the particular impact that negative publicity has upon disadvantaged women. This group was presented as being particularly likely to be confused by public debates, and vulnerable to screening disengagement:

*“There’s probably people in the* [suburbs of lower socioeconomic status] *who stop going to screening. Because they’re not as sophisticated … and they come from non-English speaking backgrounds. The message they get is that screening is not needed… It’s okay if you’re in the* [suburbs of higher socioeconomic status] *because you’ll keep coming anyway.”* (Expert #29, clinician)

In this frame, appropriate solutions focussed on preventing a fall in participation rates. They included: avoiding any implication that overdiagnosis is a harm; keeping discussions confined to academic circles; and informing women about overdiagnosis only when attendance is secured (such as at the point of mammogram or after diagnosis).

### Frame 3: don’t hide the overdiagnosis problem from women

*“We should absolutely tell people, ‘These are the benefits, these are the harms’; and some people say that public health benefits should be what we are aiming for, but for me I think you absolutely cannot compromise on telling people. It’s just not something I’m prepared to do.”* (Expert #23, researcher NOS)

This frame centres on the lack of communication about overdiagnosis from screening providers to women. Experts acknowledged that while some women prefer a simple advisory message about breast screening, others want an informed decision making process, with the readily available and easily-understood information. The current lack of communication about overdiagnosis was presented as a deliberate strategy by screening providers to avoid risking a decline in participation. In this frame, informed choice was an absolute right for individual women, taking priority over the delivery of population health benefits.

The solution was to make information about overdiagnosis available to women, despite the inherent complexities in the topic and the tension with trying to encourage participation:

*“I agree with you that the experts can’t agree and how do you talk to women about it, and it is a very complex area and hard to talk about, but clearly an important issue in the context of screening… I think you have to share with women your uncertainty.”* (Expert #25, epidemiologist)

This frame accommodated a variety of solutions ranging from detailed publicising of overdiagnosis information in every screening pamphlet and advertisement, to making detail of possible harms from screening available upon request. In this frame provision of information could co-exist alongside government promotion of screening.

### Frame 4: we need to know the overdiagnosis rate

*“There is a recognition that there are tumours found that are either frankly non-progressive or are likely to progress so slowly they don’t matter. I don’t think too many people would say, ‘Well that wouldn’t exist at all’. The argument is over how much and the scale of that.”* (Expert #22, epidemiologist)

In this frame, the main problem was overdiagnosis measurement and quantification. Experts spoke of overdiagnosis as being of indeterminate significance because of uncertainty about the overdiagnosis rate. They saw the wide range of estimates as a central conundrum, possibly explainable by different methodologies and variable data sets. A subsidiary problem was the inconsistent presentation of overdiagnosis figures, variably portrayed as acceptably low by comparing with the (large) number of cancers diagnosed, or as unacceptably high by comparing with the (smaller) number of lives saved by screening. This made it difficult to compare studies and understand the implications of overdiagnosis. In this frame sloppy research methods aimed at generating quick or provocative publications were a particular problem, eliciting strong disapproval. The first step to solving this quantitative problem would be to reach consensus on the most reliable and robust ways to calculate and present overdiagnosis.

### Frame 5: balancing harms and benefits is a personal matter

*“Descriptively they’re quite different … I don’t think there is any formula for the balance… It’s very subjective of the balance of disparate outcomes.”* (Expert #20, clinician)

Through this frame, the problem was comparing harms and benefits of breast screening. Experts discussed both overdiagnosis harms and mortality benefits accruing from breast screening. They suggested that while each are likely to be important to women, current estimates about their rates meant that harms and benefits were closely balanced; in this situation, qualitative differences between the two made it impossible for experts to draw exact conclusions about where and when equipoise arose. In this frame, such uncertainty required that the public should assist with decision making. Experts explained that since individual attitudes to harms and benefits would determine what was perceived as the net outcome of screening, the process of decision making needed consumer input: it was insufficient to rely on pre-determined program values or system priorities. The frame encompassed two possible solutions. Some experts discussed seeking public assistance with decision making at the policy level, using a deliberative process such as a citizens’ jury to make a ruling about the balance between benefits and harms:

*“I believe that for a lot of screening things there should be a community jury. There are some things that are obvious, that we can just proceed with them, but other things where there’s a balance between the benefits and harms, I think we need some sort of deliberative democracy process.”* (Expert #21, researcher NOS)

Others spoke of more explicit attempts to achieve informed consumer decision making, encouraging women to consider the net value of screening for themselves as individuals. They suggested screening participation decisions should be based on women’s personal priorities rather than potentially coercive input from screening providers.

### Frame 6: the problem is overtreatment

*“I don’t really believe in overdiagnosis as such. I mean, I think there’s over treatment … Finding it is not the issue. Treating – how it’s treated is the issue, as I see it.”* (Expert #9, clinician and provider)

The final frame through which overdiagnosis was understood purposefully separated the treatment process from the screening process, and presented the problem as arising from treatment decisions. Several causal elements for the growing problem of overtreatment were presented: some experts spoke of the increasing sensitivity of radiological equipment, meaning that more and more lesions were identified. Others noted that diagnostic criteria for certain pathological entities were vague, and *“not … easy to get inter-observer agreement on.”* (Expert #28, clinician) They discussed resulting disagreements about the threshold for atypia, with tendencies amongst some pathologists for ‘overcalling’ cancer so that benign changes were more likely to be named and treated as borderline lesions. Finally, experts commented on the limited research around natural history and management guidelines for low-risk lesions. Expert #28, (clinician) noted that, *“a lot of those guidelines are based on reviews of data which are not robust”* and suggested that they were instead driven by clinicians’ observer bias and accepted by women with high levels of anxiety and fear. Women with low-risk lesions were perceived as undergoing aggressive treatments while, *“you really wonder whether any of it was actually necessary.”* (Expert #13, clinician)

In this frame, both mortality benefit and harm avoidance were valued. Thus appropriate solutions in this frame maintained current screening parameters, and only altered downstream elements. Experts presented a range of solutions including: regular pathology updates on diagnostic criteria and thresholds; research into better prognostic tools (such as biological markers of aggression); development of more targeted / less harmful therapies, research into less aggressive treatment regimes for low-risk lesions; and patient-centred care for women with borderline lesions, relying on correlation between clinical, radiological and pathological findings to make a diagnosis and plan the management, rather than following set guidelines.

### How experts used frames

Each expert used between one and four frames. Some experts employed two or more moderately incommensurable frames, and were often conscious of inherent contradictions. For example Expert #7 (clinician) used both the “stop squabbling in public” and “stop hiding the problem” frames, acknowledging the possible inconsistency of this position. However, none of the experts’ discussions combined frames that were strongly incommensurable, for example, no experts used both the “overdiagnosis is harming women” and the “stop squabbling in public” frames. The “stop hiding the problem” frame was the most commonly used, and was adopted by experts working across all roles except consumer representation/advocacy. All (three) consumers working in advocacy roles used the “stop squabbling in public” frame.

There were observable patterns between experts’ overall views on breast screening and their use of overdiagnosis frames. All experts who were critical of breast screening used the “don’t hide the problem” frame, and none of them used the “stop squabbling in public” frame. Experts who were supportive of breast screening used one or other, but not both, of these frames (in approximately equal numbers), and were the only group to use the “stop squabbling in public” frame. Further detail on this is available in Additional file 2: Table S1-S2).

## Discussion

It is recognised in the breast screening literature that experts hold differing opinions about overdiagnosis, but the basis for those differences has not been explored. We identified six overdiagnosis frames in use by Australian breast screening experts and analysed the elements of each frame. There was considerable variation between frames, in terms of: how overdiagnosis was problematised, what information was highlighted as being relevant, what values were prioritised as being important, and what solutions were suggested. These multiple points of difference explain much of the controversy and disagreement that surrounds this important topic.

To our knowledge, there has been no detailed empirical study on what and how breast screening experts think about overdiagnosis. Some journals have presented debates containing opposing arguments as a way of exploring some of the diversity within this topic [25, 26]. Others have published letters to the editor in response to controversial elements within breast screening articles [27]. Our work builds upon and extends the existing literature, providing a comprehensive analysis of the frames used to talk about and understand overdiagnosis in breast screening. Previous research has suggested that consumers are largely unaware about overdiagnosis [12], but nevertheless an important avenue for future research would be to investigate whether women have pre-existing ideas and concerns about aspects of overdiagnosis that have not been captured within the frames presented here.

An understanding of the elements within different overdiagnosis frames will help those who work in, or consider participating in breast screening [28, 29]. The different frames may be a useful scaffold upon which to generate thoughtful discussion amongst practitioners. These frames also offer new tools for experts to clarify their own positions and to understand the opinions of others on overdiagnosis including views on whether and how it is a problem, and what solutions might be appropriate. This may facilitate recognition of points of agreement and form a basis for co-operative dialogue in the best interests of consumers [19]. Policy makers are faced with a baffling array of suggestions about what, if anything, should be done with regard to breast screening overdiagnosis. The experts who participated in this study offered a range of solutions, focusing on different points along the screening journey, including primary research, evidence translation and presentation, communication with consumers, screening practices, diagnostic practices, and treatment. By viewing these solutions in connection with the frame to which they belong, it becomes easier to see why one solution might be preferred over another, and by whom. Any management plan or policy is likely to need multiple solutions, and incommensurability between some frames will necessitate compromises and negotiations.

This study benefits from the open qualitative methodology, which allowed us to explore a topic about which there was little pre-existing knowledge. We were able to access the views and opinions from a range of influential individuals and expert stakeholders from different parts of Australia. Its strength lies in the depth of its enquiry and its ability to capture the complexity of the evidence base and value judgements underlying the range of different views. As with much qualitative work, we cannot make any predictions about the prevalence or pattern of our results within the wider population, and this may be a useful avenue for future survey research. While this study was limited to the Australian setting, much of the developed world has organised breast screening programs, comparable values, and access to the same body of scientific evidence, and thus the findings are likely to be broadly applicable across these countries. It is possible that experts who participated in our study were somehow different from those who were invited but did not participate. We sought to minimise any bias of this sort by ensuring that we interviewed experts with a range of attitudes to screening, and a wide variety of professional roles and experience.

## Conclusions

Our results demonstrate that experts approach overdiagnosis in various ways, see a range of issues and values at stake, and are inclined to promote different solutions. This may be an important contributor to the ongoing controversy in this topic, and offers a new explanation for why some debates about overdiagnosis are so heated. The concept of experts using different frames when thinking and talking about overdiagnosis might be a useful tool for those who are engaged in the topic, assisting with communication and facilitating better understanding of others’ viewpoints.

## Additional files

Additional file 1: **Sample interview introduction and questions (note: this list is provided as a guide only; the questions were modified to suit the experience and perspective of the interviewee).** (DOC 30 kb)
Additional file 2: **Table S1.** Overdiagnosis frames used by experts (organised according to main professional role). **Table S2.** Overdiagnosis frames used by experts (organised according to attitude to breast screening). (DOCX 35 kb)
