# Supplementary material for: Framing overdiagnosis in breast screening: a qualitative study with Australian experts
Source: BMC Cancer. 2015 Aug 28;15:606. doi: 10.1186/s12885-015-1603-4 (PMC4552426; doi:10.1186/s12885-015-1603-4)
Supplement: Additional file 1: — Sample interview introduction and questions (note: this list is provided as a guide only; the questions were modified to suit the experience and perspective of the interviewee). (DOC 30 kb) [file 12885_2015_1603_MOESM1_ESM.doc]

**Additional file 1**

*Sample interview introduction and questions (note: this list is provided as a guide only; the questions were modified to suit the experience and perspective of the interviewee)*

Thank you for agreeing to participate in this study. As you know, there has been quite a lot written in the literature and in the media about breast screening and what the program should look like. Plenty of people are happy with things the way they are, but others are not. So I’m interested in exploring that range of opinion, particularly amongst people who work in the field, including those who work in clinical practice, research, administration, or in breast cancer advocacy.

- Can you describe the scope of your professional activities that involve breast screening, to give me an idea about your involvement in the program?
- Would you like to see any changes to the current program?
  - *Prompt*: What would your ideal program be?
- There are many different ideas about breast cancer screening. Can you comment on these?
  - *Prompt*: There are some who hold very extreme views about breast cancer screening. How do you respond to these ideas? What do you think drives those views?
- *(If the topic hasn’t yet surfaced)* Recent studies suggest that some cancers found at screening would never have come to clinical attention in that person’s lifetime; for example, Marmot and colleagues suggest that for every 1 life saved by breast screening there are 3 cancers overdiagnosed. What are your thoughts on this issue?
  - *Prompt:* eg is it – non-existent; existing but not a problem ; a problem
- *(if appropriate to expert’s views)* What level of overdiagnosis do you work with?
- *(if considered a problem)* Should screening programs take any responsibility for reducing overdiagnosis? E.g. should we tailor the program to minimize overdiagnosis?
- *(if expert talks about the development of biomarkers or other prognostic tools as a way of addressing concerns about overdiagnosis)* What should we do in the meantime?
