# Supplementary material for: Framing overdiagnosis in breast screening: a qualitative study with Australian experts
Source: BMC Cancer. 2015 Aug 28;15:606. doi: 10.1186/s12885-015-1603-4 (PMC4552426; doi:10.1186/s12885-015-1603-4)
Supplement: Additional file 2: — Table S1. Overdiagnosis frames used by experts (organised according to main professional role). Table S2. Overdiagnosis frames used by experts (organised according to attitude to breast screening). (DOCX 35 kb) [file 12885_2015_1603_MOESM2_ESM.docx]

**Additional file 2**

**Table 3. Overdiagnosis frames used by experts (organised according to main professional role)**

| Expert profession^#^ | Frame 1: overdiagnosis is harming women | Frame 2: stop squabbling in public about overdiagnosis | Frame 3: don't hide the overdiagnosis problem from women | Frame 4: we need to know the overdiagnosis rate | Frame 5: balancing harms and benefits is a personal matter | Frame 6: the problem is overtreatment |
| --- | --- | --- | --- | --- | --- | --- |
| clinician |  |  | x |  |  |  |
| clinician |  |  | x |  |  |  |
| clinician |  |  | x | x |  | x |
| clinician |  |  | x |  |  |  |
| clinician |  |  | x |  |  |  |
| clinician |  |  | x |  |  | x |
| clinician | x |  | x |  |  |  |
| clinician |  |  |  |  |  | x |
| clinician |  |  |  | x | x |  |
| clinician |  |  | x |  |  |  |
| clinician |  |  | x |  |  |  |
| clinician |  |  | x |  |  |  |
| clinician |  |  | x |  |  |  |
| clinician |  | x |  |  |  |  |
| clinician |  | x |  |  |  | x |
|  |  |  |  |  |  |  |
| epidemiologist* | x |  | x | x |  | x |
| epidemiologist* |  |  | x |  | x |  |
| epidemiologist* |  |  | x | x |  |  |
| epidemiologist* | x |  | x |  |  |  |
| epidemiologist* |  |  | x | x |  |  |
| epidemiologist* |  |  | x |  |  |  |
| epidemiologist* |  |  |  | x |  |  |
| epidemiologist* |  | x |  |  |  |  |
|  |  |  |  |  |  |  |
| researcher^ | x |  | x |  | x |  |
| researcher^ |  |  |  |  |  |  |
| researcher^ |  |  | x |  |  |  |
| researcher^ |  |  |  |  |  |  |
| researcher^ |  | x |  |  |  |  |
|  |  |  |  |  |  |  |
| consumer advocate^#^ |  | x |  |  |  |  |
| consumer advocate |  | x |  |  |  |  |
| consumer advocate |  | x |  |  |  | x |
|  |  |  |  |  |  |  |
| administrator |  | x |  |  |  |  |
| administrator |  |  | x |  |  |  |

^#^Experts who held more than one professional role are classified under their main role *this category includes epidemiologists and biostatisticians; ^this category includes all non-clinical researchers who are neither epidemiologists nor biostatisticians; ^#^clinicians and researchers who worked in advocacy are categorised under their main role**Table 4. Overdiagnosis frames used by experts (organised according to attitude to breast screening)**

| Expert’s public position on breast screening | Frame 1: overdiagnosis is harming women | Frame 2: stop squabbling in public about overdiagnosis | Frame 3: don't hide the overdiagnosis problem from women | Frame 4: we need to know the overdiagnosis rate | Frame 5: balancing harms and benefits is a personal matter | Frame 6: the problem is overtreatment |
| --- | --- | --- | --- | --- | --- | --- |
| supportive |  |  | x |  |  |  |
| supportive |  | x |  |  |  |  |
| supportive |  | x |  |  |  |  |
| supportive |  | x |  |  |  |  |
| supportive |  | x |  |  |  | x |
| supportive |  | x |  |  |  |  |
| supportive |  |  | x | x |  |  |
| supportive |  |  | x |  |  |  |
| supportive |  |  | x |  |  |  |
| supportive |  |  |  | x | x |  |
| supportive |  |  | x |  | x |  |
| supportive |  |  | x |  |  |  |
| supportive |  |  | x |  |  |  |
| supportive |  |  | x |  |  |  |
| supportive |  | x |  |  |  |  |
| supportive |  | x |  |  |  | x |
| mostly supportive |  |  | x |  |  |  |
| mostly supportive |  |  | x | x |  | x |
| mostly supportive |  |  | x |  |  |  |
|  | | | | | | |
| critical | x |  | x |  |  |  |
| critical | x |  | x |  | x |  |
| critical |  |  | x | x |  |  |
| critical | x |  | x |  |  |  |
| critical | x |  | x | x |  | x |
|  | | | | | | |
| unknown |  |  | x |  |  |  |
| unknown |  |  | x |  |  | x |
| unknown |  |  |  |  |  | x |
| unknown |  |  |  | x |  |  |
| unknown |  |  |  |  |  |  |
| unknown |  |  | x |  |  |  |
| unknown |  |  |  |  |  |  |
| unknown |  | x |  |  |  |  |
| unknown |  |  | x |  |  |  |
